# Care at a non-university hospital: an independent risk factor for mortality in ARDS?

**DOI:** 10.1186/s13054-017-1778-y

**Published:** 2017-07-31

**Authors:** Bourke Tillmann, Hannah Wunsch

**Affiliations:** 1 Department of Critical Care Medicine, Sunnybrook Health Sciences Centre, 2075 Bayview Ave., Room D1.08, Toronto, Ontario M4N 3M5 Canada; 2 Interdepartmental Division of Critical Care Medicine, University of Toronto, Toronto, Ontario Canada; 3 Department of Anesthesia, University of Toronto, Toronto, Ontario Canada; 4 Department of Anesthesiology, Columbia University, New York, NY USA

Many studies over the past 20 years have found an association between receiving treatment at a university or teaching hospital and decreased hospital mortality [1–3]. However, few studies have assessed this question specifically among critically ill patients [4]. Recently, a study by Raymondos and colleagues examined the relationship between care in a university hospital and mortality for patients with the acute respiratory distress syndrome (ARDS) [5]. The study was a sub-analysis of a prospective, observational cohort of patients with respiratory failure (Second VENTILA study) [6]. The authors found that, although the characteristics of ARDS patients were somewhat similar between hospitals, unadjusted hospital mortality was significantly higher in non-university hospitals compared with university hospitals (57.5 versus 39.3%; absolute difference of 18.2%, *p* = 0.012). This difference remained after adjustment for patient factors, as well as factors related to individual patient management, complications during ventilation, and hospital characteristics (odds ratio 2.89; 95% confidence interval 1.31–6.38). Furthermore, they found a 9.6% increase in hospital mortality for ventilated patients who were not diagnosed with ARDS.

Previous studies examining the care of surgical patients have hypothesized that the presence of residents in the operating room may lead to higher mortality, but have often found either similar or better mortality [3]. Often this lower mortality is attributed to the volume of cases at these centers [3]. In the study by Raymondos et al. the university hospitals were much bigger, with more hospital beds and more ICU beds per unit, but they were not able to show a relationship between volume of ARDS patients in each hospital and outcomes. However, it is important to note that many hospitals in the study only had one or two ARDS patients during the one-month study period.

In addition to case volume, other factors may be important in the care of critically ill patients. For instance, implementation of recent, accepted best practice may be different between university and non-university hospitals. In the CESAR study, a randomized controlled trial of transfer to regional extracorporeal membrane oxygenation (ECMO) centers, one hypothesis for the better outcomes for these patients was better adherence to overall best care practices, including low tidal volume ventilation [7]. However, data from Germany assessing ICU care for sepsis patients found that university hospitals had similar rates of compliance with guidelines compared with other hospitals, despite reporting higher adherence [4].

Staffing in ICUs may also be very different in university hospitals. Depending on the system, this may include an overall higher physician to patient ratio due to the presence of trainees on the team, increased access to specialists, and/or more involvement of multidisciplinary team members, such as pharmacists on rounds. While the presence of trainees and specialists will certainly be increased at academic centers worldwide, and multidisciplinary care teams are more frequent in ICUs in teaching hospitals in the US [8], their presence elsewhere is not well described. Another challenge in comparing the outcomes for patients at university and non-university settings is the practice of transferring patients who are very sick but considered salvageable to larger, academic centers [9]. In the study by Raymondos et al., it is unclear how such transfers were accounted for. However, every hospital system has either formal or informal transfer networks [10]. Such transfers to tertiary care centers may skew reported outcomes in the different locations.

Finally, the patient and family preferences for care may be different in university and non-university settings. It is possible that (when there is an option for location of care) patients and families who prefer more aggressive care tend to choose university hospitals while those who are less focused on such issues may tend to stay in non-university settings [11]. The results by Raymondos et al. were striking for the very different lengths of mechanical ventilation, different rates of tracheostomy, and yet similar mortality rates in the ICU. The authors speculate that the shorter duration of mechanical ventilation in non-university hospitals may lead to more deterioration on the ward. However, it is also possible that patients and families opted for fewer tracheostomies and more palliation. In a recent analysis of nighttime discharges in Australia, such differences in care preferences were found to explain much of the higher mortality seen for patients discharged at night [12]. More and more, information on care preferences will be important for interpretation of mortality results in critically ill patient populations [13].

The factors that account for lower hospital mortality in ARDS patients at university hospitals represent a complex matrix, and may not be ubiquitous across university hospitals, or limited to these centers. However, regardless of the underlying cause of the lower mortality seen at specific centers, one question needs to be addressed: how do we identify best care models, particularly in the complex world of the ICU? One response to improved mortality demonstrated at specific centers is the creation of regional health care systems, as is common in trauma [14]. However, the creation of these centers is a mammoth undertaking requiring input from politicians, health care providers, and administrators [15]. In addition, the creation of large interconnected health care systems needs to be based on the best possible evidence. Further studies are needed to delineate which patients may benefit from receiving their therapy at specific hospitals, how care differs in these centers, and how to identify them.

## Abbreviations

ARDS: Acute respiratory distress syndrome
ECMO: Extracorporeal membrane oxygenation
ICU: Intensive care unit.
